# Glyoxalase 1 gene improves the antistress capacity and reduces the immune inflammatory response

**DOI:** 10.1186/s12863-019-0795-z

**Published:** 2019-12-10

**Authors:** Fukuan Du, Yan Li, Jing Shen, Yueshui Zhao, Parham Jabbarzadeh Kaboli, Shixin Xiang, Xu Wu, Mingxing Li, Jiangyao Zhou, Yuan Zheng, Tao Yi, Xiang Li, Jing Li, Zhangang Xiao, Qinglian Wen

**Affiliations:** 1 Laboratory of Molecular Pharmacology, Department of Pharmacology, School of Pharmacy, Southwest Medical University, Luzhou, Sichuan China; 2 South Sichuan Institute of Translational Medicine, Luzhou, Sichuan China; 3 Sichuan Neijiang Medical School, Neijiang, Sichuan China; 4 Neijiang Health and Health Vocational College, Neijiang, Sichuan China; 5 School of Chinese Medicine, Hong Kong Baptist University, Hong Kong, China; 6 Department of Oncology and Hematology, Hospital (T.C.M) Affiliated to Southwest Medical University, Luzhou, Sichuan China; 7 Department of Oncology, Affiliated Hospital of Southwest Medical University, Luzhou, Sichuan China

**Keywords:** *Coilia nasus*, Glyoxalase 1 gene, Immunity, Inflammation, Oxidative stress, Stress

## Abstract

**Background:**

Fish immunity is not only affected by the innate immune pathways but is also triggered by stress. Transport and loading stress can induce oxidative stress and further activate the immune inflammatory response, which cause tissue damage and sudden death. Multiple genes take part in this process and some of these genes play a vital role in regulation of the immune inflammatory response and sudden death. Currently, the key genes regulating the immune inflammatory response and the sudden death caused by stress in *Coilia nasus* are unknown.

**Results:**

In this study, we studied the effects of the *Glo1* gene on stress, antioxidant expression, and immune-mediated apoptosis in *C. nasus*. The full-length gene is 4356 bp, containing six exons and five introns. Southern blotting indicated that *Glo1* is a single-copy gene in the *C. nasus* genome. We found two single-nucleotide polymorphisms (SNPs) in the *Glo1* coding region, which affect the three-dimensional structure of Glo1 protein. An association analysis results revealed that the two SNPs are associated with stress tolerance. Moreover, *Glo1* mRNA and protein expression of the heterozygous genotype was significantly higher than that of the homozygous genotype. Na^+^ and sorbitol also significantly enhanced *Glo1* mRNA and protein expression, improved the fish’s antioxidant capacity, and reduced the immune inflammatory response, thus sharply reducing the mortality caused by stress.

**Conclusions:**

Glo1 plays a potential role in the stress response, antioxidant capacity, and immune-mediated apoptosis in *C. nasus*.

## Background

The estuarine tapertail anchovy, *Coilia nasus*, is a commercially important species in China because of its nutritive value and delicacy [1]. The fish is widely distributed in the Yangtze River, the coastal waters of China and Korea, and the Ariake Sound in Japan [2]. *C. nasus* is an excellent model animal for stress research because it is highly responsive to stress. Transport and loading the fish often induces stress and this stress response can cause sudden death [3], a phenomenon that also occurs in humans [4]. Currently, the key genes regulating stress-induced sudden death in *C. nasus* are unknown. Therefore, this topic warrants further study.

The glyoxalase system catalyzes the conversion of reactive acyclic α-oxoaldehyde into the corresponding α-hydroxyacid [5, 6]. This system involves two enzymes, glyoxalase 1 (Glo1) and glyoxalase 2 (glo2), and a catalytic amount of reduced glutathione (GSH) [7]. Glo1 is the rate-limiting enzyme in this system and it catalyzes the isomerization of the hemithioacetal that forms spontaneously in the conversion of α-oxoaldehyde and GSH to S-2-hydroxyacylglutathione derivatives, which reduces the steady-state concentrations of physiological α-oxoaldehyde and the associated glycation reactions [8–10]. Glo1 reportedly plays an important role in many diseases [11–15], including diabetes, in which this gene is suppressed [16]. Furthermore, Glo1 suppression has also been linked to the development of the vascular complications of diabetes and also to nephropathy, retinopathy, neuropathy, and cardiovascular disease [17–19]. Increasing Glo1 activity is important in the treatment of diabetes and these complications [20].

In our previous studies, the stress response in *C. nasus* induced hyperglycemia, which induced oxidative stress, activated the immune inflammatory response, and caused tissue damage [3, 21]. As we reported, Glo1 alleviated this hyperglycemia-induced damage. Therefore, in this study, we investigated whether Glo1 is associated with sudden death in *C. nasus*.

## Results

### Sudden death caused by stress and *Glo1* gene response in *C. nasus*

**Fig. 1:**
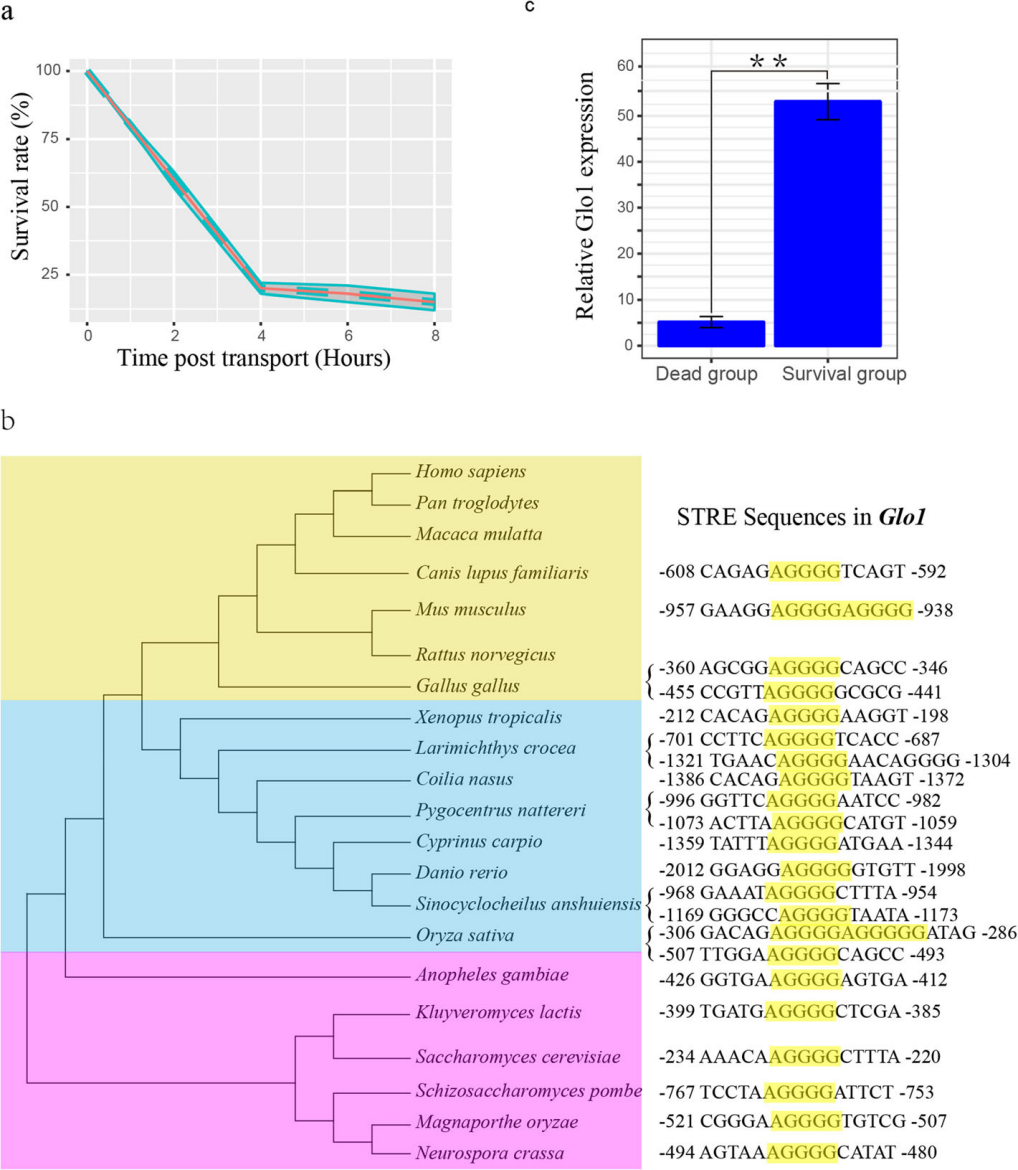
Transport stress and *Glo1* response in *C. nasus*. (**a**) Changes in the survival rate after transport stress. (**b**) Stress response elements (SREs) in the *Glo1* genes of different species. (**c**) *Glo1* mRNA expressions in the dead fish and surviving fish groups

One hundred eighty individuals from a random population were used in the transport experiment. The results showed that the survival rate decreased sharply at 0–4 h, declined gradually after 4 h, and then became slight after 6 h. Of the initial fish population, 12% were still alive after 8 h (Fig. 1a). These data indicate that individual fish showed different stress tolerance, and this difference arose from genetic differences, which could include DNA variation and epigenetics. However, the genes related to sudden death in *C. nasus* were unknown.

Our previous studies have shown that oxidative stress is a major cause of stress damage in *C. nasus*, so we speculated that the stress-induced sudden death gene/s in this fish should meet two basic conditions: (i) they are involved in oxidative stress; and (ii) they should contain stress response elements (SREs). Based on these two conditions, we identified the *Glo1* gene. The sequences of this gene have been reported in humans [22], mammals [23–26], and fish [27, 28], and its function is conserved. More importantly, SREs have been found in the *Glo1* 5′ untranslated regions (UTRs) in these species (Fig. 1b). We determined the expression levels of *Glo1* in the brains of the surviving and dead *C. nasus* with RT–qPCR. The expression of *Glo1* was significantly higher in the surviving group than in dead group (Fig. 1c). These results indicate that this gene is regulated by stress.

### *Glo1* gene copies in the *C. nasus* genome

**Fig. 2:**
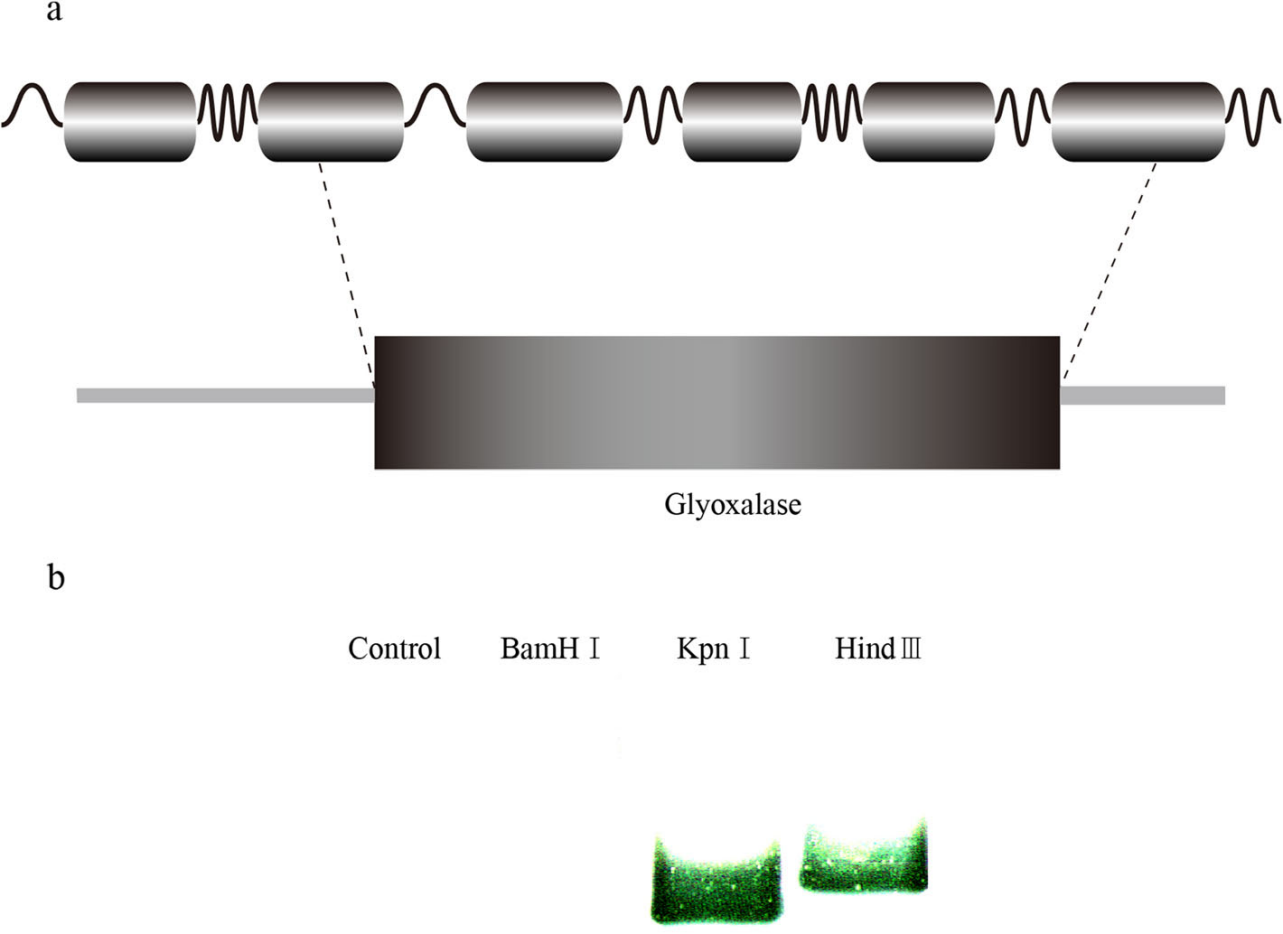
Schematic diagram of the *Glo1* gene structure and Southern blotting results. (**a**) Schematic diagram of the *Glo1* gene structure. The full-length gene is 4356 bp long, with six exons and five introns. (**b**) Southern blotting results for *Glo1* in *C. nasus*

To clarify the correlation between *Glo1* gene expression and sudden death, we determined the full DNA sequence of the *Glo1* gene. The full-length gene is 5274 bp long, and contains six exons and five introns (Fig. 2a, GenBank accession number: MK116541). The copy number of *Glo1* in the *C. nasus* genome was determined with Southern blotting, which showed a single insertion site for *Glo1* in the *C. nasus* genome (Fig. 2b).

### Association of *Glo1* gene alleles with stress

**Fig. 3:**
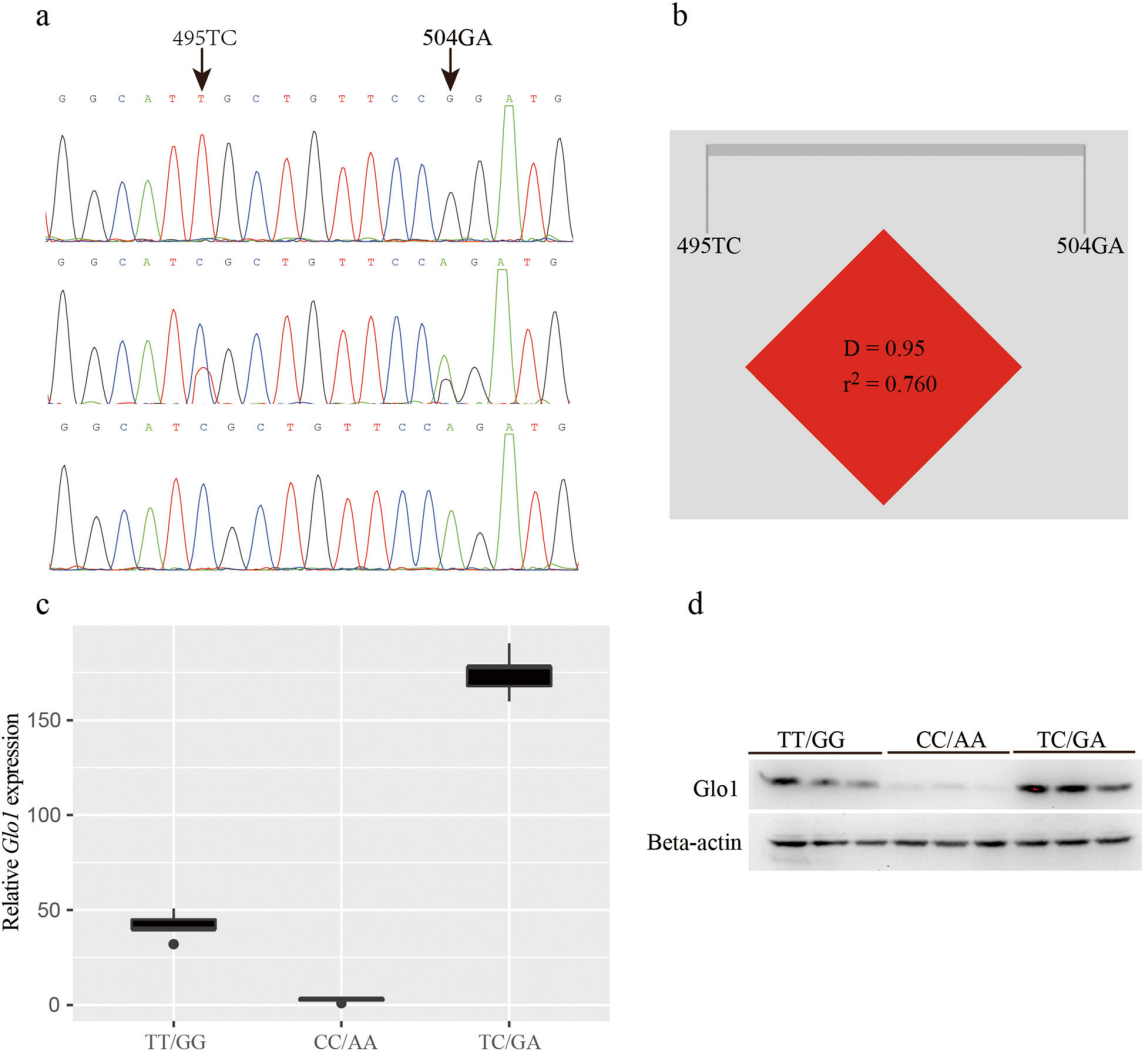
Association analysis of *Glo1* polymorphism and stress tolerance. (**a**) Two polymorphic loci (495 T/C and 504 G/A) in the *Glo1* gene. (**b**) Analysis of paired-locus linkage disequilibrium of 495 T/C and 506G/A. (**c**) The mRNA expressions of the heterozygous genotype (TC/GA) and homozygous genotypes (CC/AA, TT/GG). (**d**) Protein expressions of the heterozygous genotype (TC/GA) and homozygous genotypes (CC/AA, TT/GG)

To determine whether the natural variation in any of the *Glo1* genes is associated with the variation in stress tolerance in *C. nasus* individuals, an association analysis was conducted for each SNP in the *Glo1* gene. We sequenced the whole gene with six pairs of primers, and two polymorphic loci (495 T/C and 504 G/A) were detected (Fig. 3a), both in the coding sequence.

We tested whether the genotypic frequencies were in Hardy–Weinberg equilibrium using the goodness-of-fit χ^2^ test. Both *P* values were > 0.05. A correlation analysis of stress tolerance and the genotype distribution was performed with R3.3.3, and the significance of the correlation was confirmed with the χ^2^ test. These results indicated that both SNPs were significantly associated with stress tolerance (both *P* < 0.05; Table 1). For SNP 495 T/C, the CC, TC, and TT genotype frequencies were 17.1, 48.6, and 34.3%, respectively, in the dead group, whereas the corresponding frequencies in the surviving group were 15.8, 78.9, and 5.3%, respectively, which were all significantly different from those in the dead group (all *P* < 0.05; Table 1). For SNP 506G/A, the AA, GA, and GG genotype frequencies were 34.3, 28.6, and 37.1%, respectively, in the dead group, whereas the corresponding frequencies in the surviving group were 15.8, 78.9, and 5.3%, respectively, which were all significantly different from those in the dead group (all P < 0.05; Table 1). These data indicate that the two SNPs are associated with stress tolerance.

**Table 1:** Association analysis of SNPs in *Glo1* that confers stress tolerance

Locus	Genotype	Dead	Survival	χ2 (P)
495 T/C	CC	6 (0.171)	3 (0.158)	6.240 (0.044)
	TC	17 (0.486)	15 (0.789)	
	TT	12 (0.343)	1 (0.053)	
504G/A	AA	12 (0.343)	3 (0.158)	13.095 (0.001)
	GA	10 (0.286)	15 (0.789)	
	GG	13 (0.371)	1 (0.053)	

An analysis of the paired-locus linkage disequilibrium revealed that SNPs 495 T/C and 506G/A were in strong linkage disequilibrium, and they were selected for a haplotype analysis (Fig. 3b). Four common haplotypes were detected in both the dead and surviving groups (global *P* = 0.152), whereas haplotype TA (495 T–506A) was only found in the dead group (8.70%, *P* = 0.06).

According to this association analysis, the stress tolerance conferred by different *Glo1* genotypes differed. Therefore, the expression of Glo1 could differ in the fish with these genotypes. To test this, we determined the *Glo1* mRNA and protein expression in both the dead and surviving fish groups. The results showed the mRNA expression of the heterozygous genotype (TC/GA) was significantly higher than that of the homozygous genotypes (CC/AA, TT/GG) (Fig. 3c). Moreover, the protein expression levels were consistent with the mRNA levels (Fig. 3d). These results indicate that level of Glo1 expression is closely associated with stress tolerance.

**Fig. 4:**
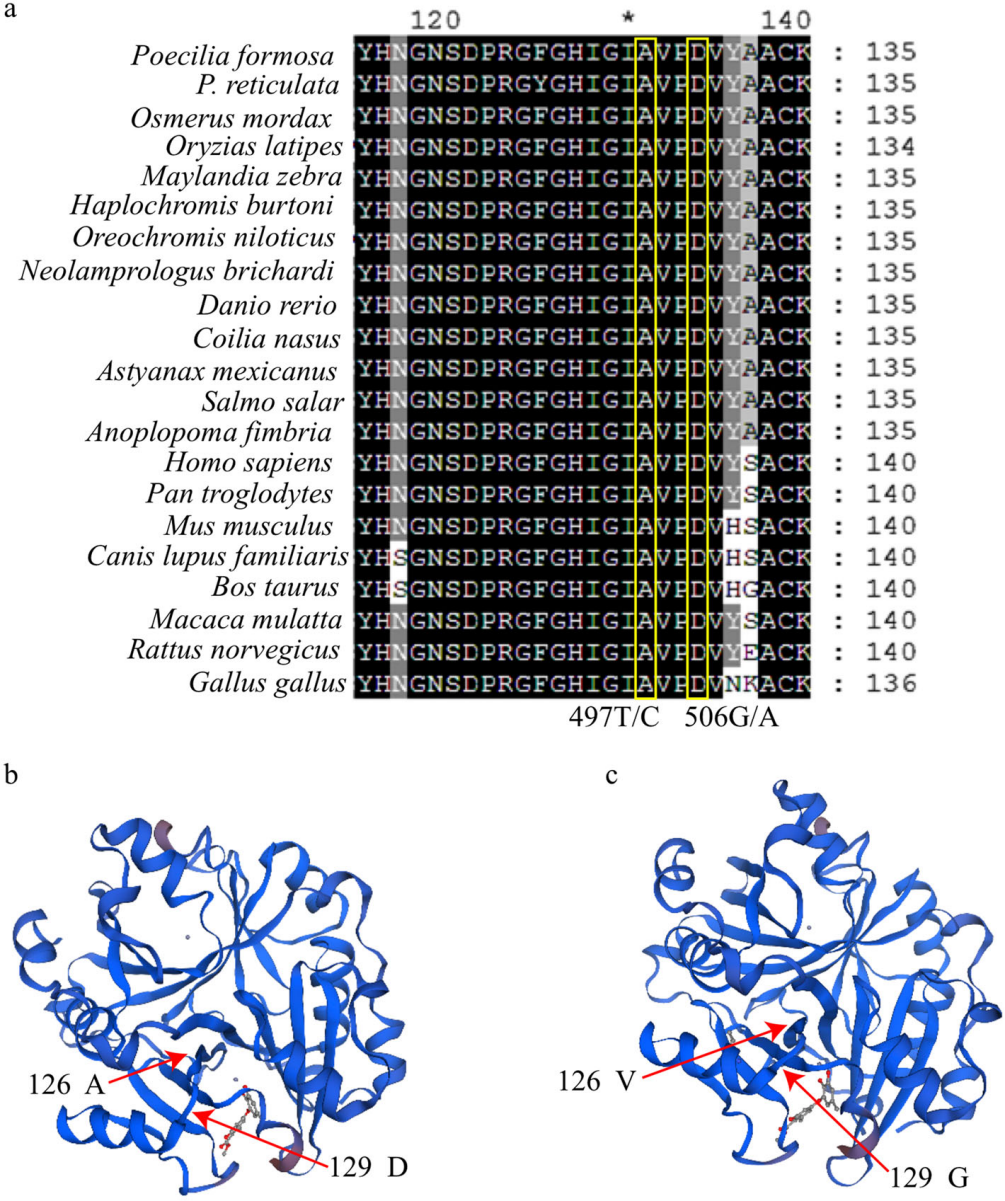
Comparison of the Glo1 amino acid sequences and three-dimensional protein structures predicted for the different Glo1 genotypes. (**a**) Comparison of the Glo1 amino acid sequences of different species. (**b**) Three-dimensional Glo1 protein structure corresponding to genotype 495 T/506G. (**c**) Three-dimensional Glo1 protein structure corresponding to genotypes 495C/506A

By comparing the Glo1 amino acid sequences of different species, we found that the two SNPs are located in conserved regions of the protein (Fig. 4a). Moreover, 495 T/C (126 A:V) and 506G/A (129 D:G) are nonsynonymous mutations (Fig. 4a). Therefore, we predicted the three-dimensional protein structures conferred by the different genotypes, and found that these mutations affected the three-dimensional structure of the Glo1 protein (Fig. 4b, c). This largely explains why these two SNPs are associated with stress tolerance.

### *Glo1* regulation and the stress survival rate

**Fig. 5:**
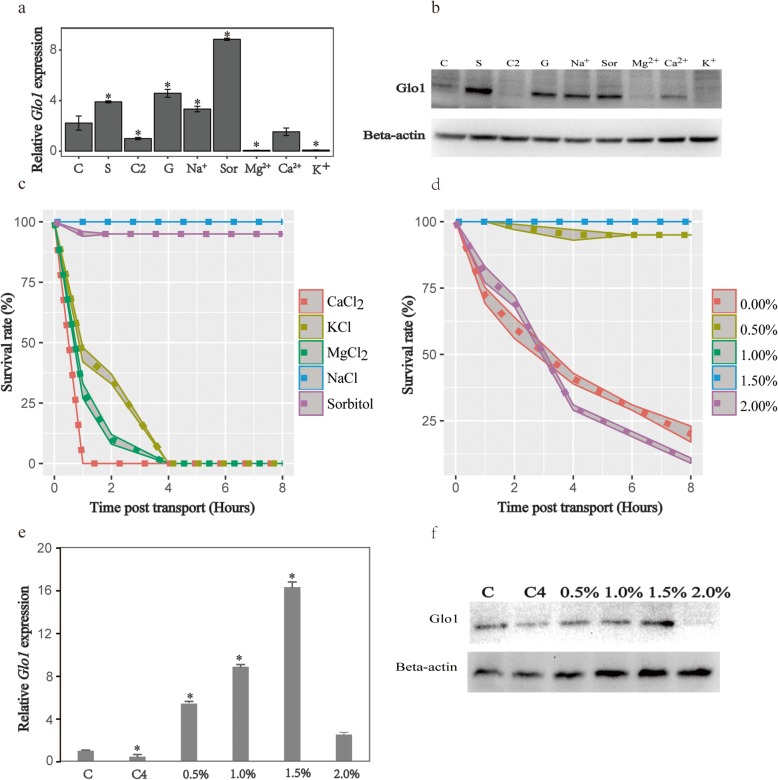
Glo1 mRNA and protein expression and the survival rate regulated by different ions. (**a**) *Glo1* mRNA expression regulated by different ions. Data are expressed as the ratio of *Glo1* mRNA expression in the brain to its expression in the C2 group (mean ± SD). Data with different superscript letters are significantly different (*P* < 0.05). C, control, no ions; S, sea salt; C2, control 2, no ions, 8 h after transport; Ga, Glo1 agonist, SEC; Na^+^, NaCl; Sor, sorbitol; Mg^2+^, MgCl_2_; K^+^, KCl; Ca^2+^, CaCl_2_. (**b**) Glo1 protein expression was regulated by different ions. (**c**) Changes in survival rate regulated by different ions. (**d**) Changes in survival rate regulated by different concentrations of NaCl. (**e**) *Glo1* mRNA expression regulated by different concentrations of NaCl. Data are expressed as the ratio of *Glo1* mRNA expression in the brain to its expression in the C group (mean ± SD). Data with ^*^ are significantly different (P < 0.05). C, no NaCl control; C4, no NaCl control after 4 h stress. The following are the 0.5, 1.0, 1.5, and 2.0% NaCl groups. (**f**) Glo1 protein expression was regulated by different concentrations of NaCl

To clarify whether the regulation of *Glo1* expression affects the survival rate of *C. nasus* after stress, the regulation of *Glo1* mRNA expression by different ions was investigated with RT-qPCR. We found that a *Glo1* agonist, S-ethyl cysteine (SEC), significantly increased the mRNA expression of *Glo1* (Fig. 5a). Moreover, seawater salts, Na^+^, and sorbitol also significantly enhanced *Glo1* mRNA expression (Fig. 5a). Among these, Na^+^ most notably enhanced *Glo1* mRNA expression. However, Mg^2+^ and Ca^2+^ also significantly inhibited *Glo1* mRNA expression (Fig. 5a). The expression of Glo1 protein was detected with western blotting, and the results were consistent with the expression of *Glo1* mRNA (Fig. 5b).

Glo1 is associated with sudden stress-associated death, so regulating the expression of *Glo1* should affect the survival rate of *C. nasus* after stress. To test this hypothesis, we injected fish with the Glo1 agonist and found that the 8-h stress-associated survival rate dropped to 58% (Fig. 5c). The addition 0.1% NaCl or sorbitol to the fish culture water increased the survival rate to more than 95%, but the effect of NaCl was most significant (Fig. 5c). To clarify the optimal Na^+^ concentration that protects *C. nasus* against stress, we tested five concentrations, and found that 1.0–1.5% NaCl resulted in the highest survival rate (Fig. 5d). These results indicate that NaCl is an ideal antistress agent for *C. nasus*. Meanwhile, the mRNA expression (Fig. 5e) and protein expression were also significantly upregulated by 1.0–1.5% NaCl.

### Changes in *Glo1* expression affect immune inflammation and antioxidant capacity

As in our previous study [3], transport and loading stress induced oxidative stress in *C. nasus*, and this oxidative stress activated the apoptosis pathway mediated by tumor necrosis factor α (TNF-α), ultimately causing tissue damage. Na^+^ and sorbitol significantly increased the expression of Glo1 and reduced the mortality caused by stress. Therefore, we speculated that Na^+^ significantly improved the antioxidant capacity and reduced the immune inflammatory response of the fish. To test this hypothesis, we detected the lipid peroxidation (LPO) levels in the fish tissues and intermediates of the apoptosis pathway. The Glo1 agonists, Na^+^, and sorbitol significantly inhibited the upregulation of all these factors (Table 2). We also detected important indicators of antioxidation, including the total antioxidant capacity (T-AOC), superoxide dismutase (SOD), catalase (CAT), and glutathione peroxidase (GSH-Px). The Glo1 regulators (SEC, NaCl, and sorbitol) significantly increased the T-AOC and GSH-Px activities, reduced the LPO content, inhibited TNF-α-mediated cellular immune inflammation, and alleviated the injury induced by stress (Table 3).

**Table 2:** Effects of *Glo1*-regulating reagents on the apoptosis pathway mediated by TNF-α

	LPO (nmol/mg)	TNFα (g/L)	Caspase 9 (IU/L)	Caspase 3 (IU/L)	Cytochrome c (nmol/L)
Control 0	0.23 ± 0.08^a^	2.47 ± 0.15^a^	36.90 ± 2.45^a^	42.66 ± 3.23^a^	123.23 ± 4.05^a^
Control 8	0.97 ± 0.06^b^	12.00 ± 0.23^b^	90.89 ± 4.22^b^	89.28 ± 3.45^b^	435.34 ± 6.38^b^
Glo 1 agonists	0.54 ± 0.07^c^	3.32 ± 0.45^c^	52.38 ± 3.13^c^	48.58 ± 6.43^a^	204.55 ± 12.45^c^
NaCl	0.46 ± 0.04^c^	2.49 ± 0.23^a^	42.89 ± 3.13^d^	49.46 ± 2.69^c^	180.43 ± 5.34^d^
CaCl_2_	1.24 ± 0.05^d^	11.56 ± 0.33^b^	89.34 ± 6.19^b^	98.33 ± 6.67^d^	590.83 ± 14.65^e^
Mg Cl_2_	0.98 ± 0.04^b^	12.03 ± 0.45^b^	106.23 ± 10.48^e^	89.45 ± 3.13^b^	467.93 ± 7.12^f^
KCl	0.73 ± 0.07^e^	5.32 ± 0.43^d^	80.78 ± 8.34^b^	56.78 ± 4.78^e^	304.43 ± 9.43^g^
Sorbitol	0.50 ± 0.06^c^	2.35 ± 0.33^a^	38.49 ± 3.13^a^	45.48 ± 7.29^a^	178.93 ± 10.82^d^

Values presented are the means of three replicates. Means in the same column with different superscript letters are significantly different (P < 0.05)

**Table 3:** Effects of *Glo1*-regulating reagents on total antioxidant capacity (T-AOC), superoxide dismutase (SOD), catalase (CAT), and glutathione peroxidase (GSH-Px)

	T-AOC (U/mg prot)	SOD (U/mg prot)	CAT(U/mg prot)	GSH-Px (U)
Control 0	61.82 ± 7.08^a^	0.64 ± 0.03^a^	10.28 ± 1.23^a^	5.50 ± 0.08^a^
Control 8	30.23 ± 4.23^b^	0.54 ± 0.09^b^	9.38 ± 2.08^a^	25.45 ± 1.23^b^
Glo 1 agonists	140.32 ± 4.32^c^	0.73 ± 0.05^c^	8.99 ± 1.56^a^	8.24 ± 0.05^c^
NaCl	163.26 ± 5.67^d^	0.63 ± 0.06^a^	10.23 ± 2.08^a^	6.38 ± 0.06^d^
CaCl_2_	15.34 ± 2.34^e^	0.45 ± 0.03^b^	10.13 ± 1.46^a^	42.34 ± 0.09^e^
Mg Cl_2_	19.32 ± 3.56^e^	0.34 ± 0.04^b^	11.34 ± 1.03^a^	26.34 ± 1.32^f^
KCL	18.23 ± 3.23^e^	0.45 ± 0.05^b^	8.92 ± 0.05^b^	24.34 ± 0.05^f^
Sorbitol	169.34 ± 2.33^d^	0.63 ± 0.34^a^	9.56 ± 0.09^a^	6.78 ± 0.07^d^

Values presented are the means of three replicates. Means in the same column with different superscript letters are significantly different (P < 0.05)

### Glo 1 expression in cancers

**Fig. 6:**
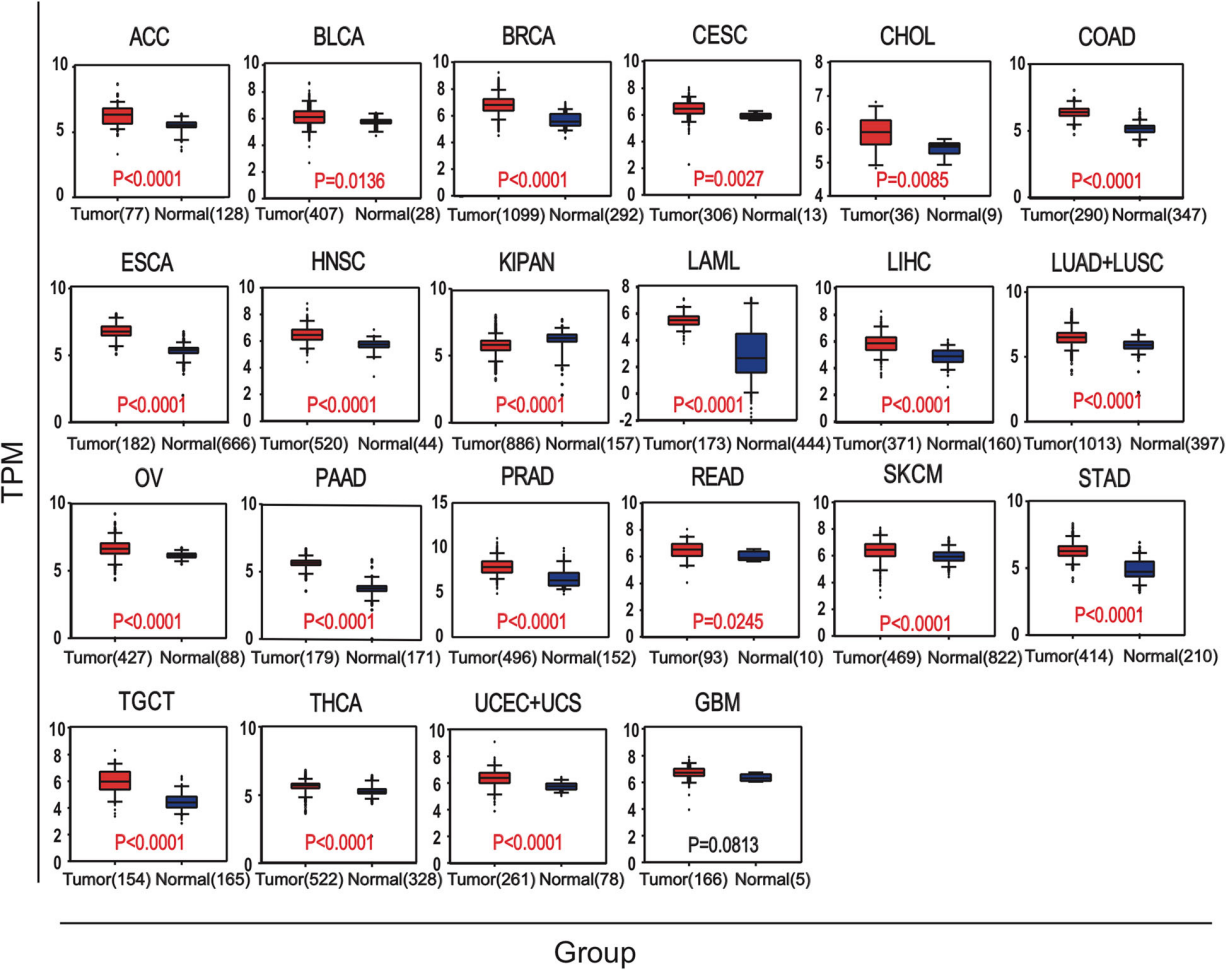
RNA sequencing data from TCGA and the GTEX database of cancer patients and normal samples were used to analyze the expression levels of *Glo 1* genes in different kinds of cancers. The number of samples is shown in the figure

**Fig. 7:**
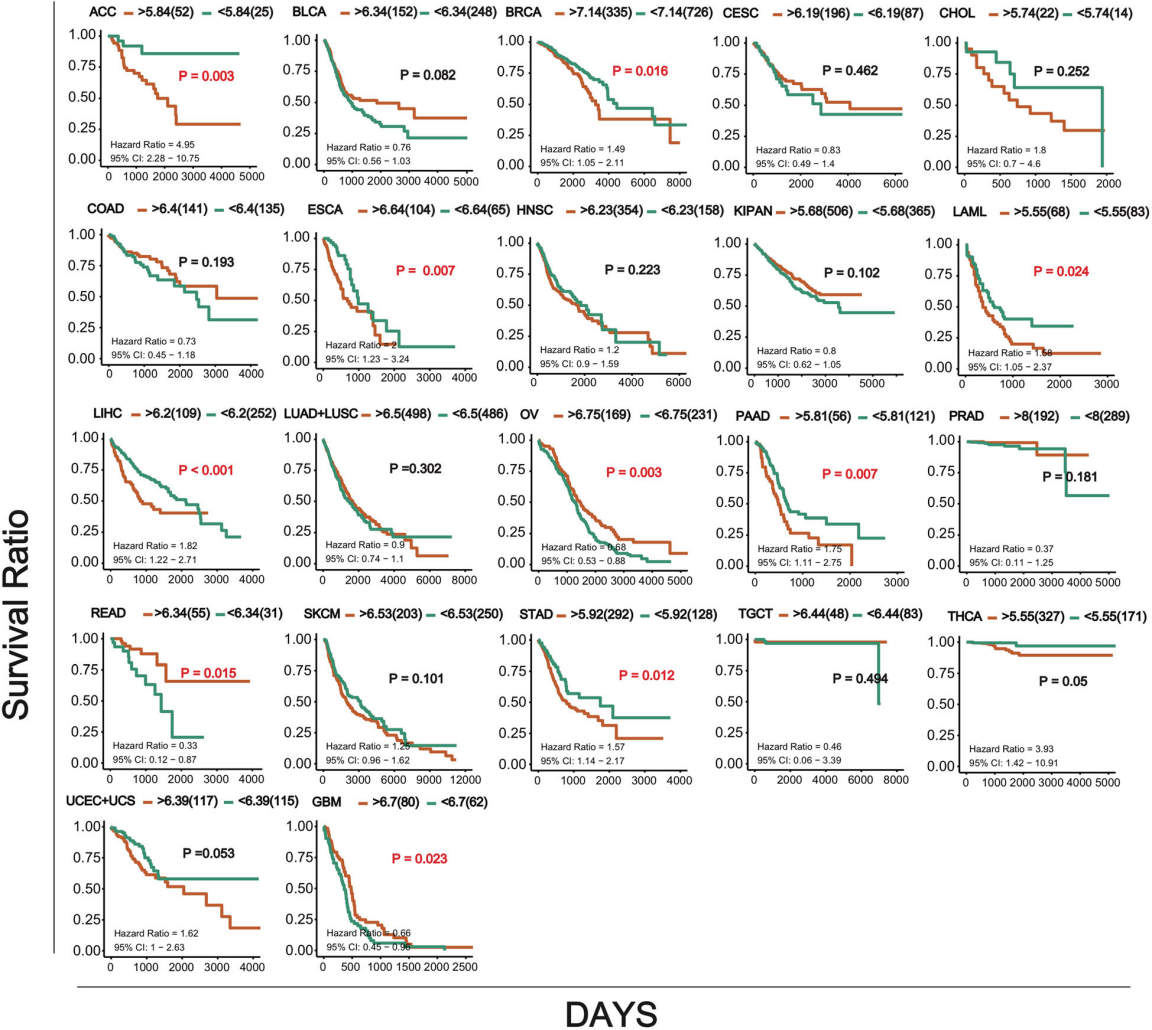
Kaplan-Meier survival curves of *Glo1* genes in different kinds of cancers, based on the expression levels. The orange lines represent high expression and the green lines represent low expression

In order to know if there was a correlation between *Glo1* and cancer, first, we compared the levels of expression between normal samples and patients with different kinds of cancers and presented them in a box plot. The results showed that except for GBM, the other cancers all showed significant differences. Most cancers were significantly upregulated in tumor samples, and in contrast, only KIPAN was significantly downregulated. We then conducted a survival analysis of the *Glo1* gene in cancers (Fig. 6). Kaplan-Meier analysis showed that about one-half of cancers having differential expression were significantly related to overall survival (OS) (Fig. 7). These results suggested that the *Glo1* gene may play a significant role in different kinds of cancers.

## Discussion

Stress is a response of organisms to the environment [29, 30]. As previously reported, the stress responses in fish usually activate factors in the hypothalamic–pituitary–adrenal axis [21, 31], releasing the stress hormone cortisol [32], which in turn regulates the metabolism of carbohydrates and lipids [33, 34], and even the immune response. Therefore, fish immunity is not only affected by its innate immune pathways but is also triggered by the stress response. In this study, we investigated the effects of *Glo1* gene expression on stress, antioxidation, and immune-mediated apoptosis in *C. nasus*. Our results show that *Glo1* expression can significantly alter immune inflammation and the expression of apoptosis-related factors, in another important mechanism of fish immunoregulation.

Glo1 is reportedly associated with many diseases, including diabetes [35], cancer [36], and depression [37, 38]. However, no association between Glo1 and sudden death has previously been reported. *Coilia nasus* is a fish with a strong stress response, and its daily management and netting often cause its sudden death [3]. Therefore, this species is an important model for the study of sudden death. In the present study, we found that specific genotypes of *Glo1* were associated with sudden death, and that both Na^+^ and sorbitol significantly increased the mRNA and protein expression of *Glo1*, which further regulated the fish survival rate after stress. It has been reported that SEC is an agonist of Glo1 [18]. In our study, SEC also significantly increased the expression of *Glo1* mRNA and protein in *C. nasus*. Interestingly, sea salts improved *Glo1* expression more significantly than SEC. However, sea salt is a mixture and it is important to know which ingredients in sea salts are most effective. Therefore, we tested Na^+^, K^+^, Mg^2+^, and Ca^2+^, and found that the effect of Na^+^ on *Glo1* expression was the most pronounced. A gradient experiment showed that Na^+^ concentrations of 1–1.5% best stimulated *Glo1* expression and best reduced the fish mortality rate after stress. We also examined whether the salts themselves or the osmotic pressure they exert regulate *Glo1* expression by investigating the regulatory effect of sorbitol on *Glo1* expression because sorbitol in water only changes the osmotic pressure and does not affect the ion concentration. Our results showed that sorbitol also significantly increased the expression of *Glo1*, indicating that osmotic pressure is the key factor regulating *Glo1* expression and *C. nasus* mortality after stress. However, Mg^2+^ and Ca^2+^ also inhibited the expression of *Glo1*.

The occurrence of cancer is closely related to immunity [39]. Because Glo1 can regulate immunity, the gene may be related to cancer. In order to verify this possibility, we analyzed *Glo 1* expression in normal and tumor tissues. The results showed that the expressions of *Glo1* in most cancer tissues were significantly higher than those in normal tissues (Fig. 6). Then, why is the *Glo1* expression level in cancerous tissues significantly increased? According to the Warburg theory [40], tumor cells use the glycolysis pathway to provide energy even under aerobic conditions. At the same time, due to the rapid proliferation of tumor cells, a large amount of energy is required, and carbohydrate catabolism is strengthened; these processes are similar to the stress response. According to our previous research, the glycolysis pathway produces energy and also produces a large amount of reactive oxygen species, which activate oxidative stress [3], and Glo1 plays an important role in the regulation of oxidative stress. Therefore, Glo1 in cancer tissues is at a high level of expression. From this perspective, Glo 1 may be a potential target for cancer therapy.

## Conclusions

In summary, in this study, we found that the *Glo1* gene is conserved in different species and that SREs occur upstream from these genes. There is a single copy of *Glo1* in the *C. nasus* genome, with two SNPs in the coding region. These cause nonsynonymous mutations that alter the three-dimensional structure of the Glo1 protein. An association analysis showed that the genotypes of the two SNPs correlate significantly with stress tolerance. RT–qPCR and western blotting showed that the expression of heterozygous *Glo1* genotypes was significantly higher than the expression of homozygous genotypes. Na^+^ and sorbitol significantly upregulate *Glo1* expression, inhibit immune inflammation, improve the fish’s antioxidant capacity, and reduce the mortality caused by stress. Our results collectively indicate that *Glo1* is a key functional gene involved in the sudden death induced in *C. nasus* by stress.

## Methods

### Ethical statement

All sample collections were performed in accordance with the Guidelines for Experimental Animals established by the Ministry of Agriculture of China (Beijing, China). The whole study was approved by the Animal Welfare Committee of China Agricultural University (permit number: SYXK 2007–0023).

### Experimental animals

*C. nasus* (average weight, 9.6 ± 1.2 g) were from our breeding base (Qiandaohu, Zhejiang, China). The fish were adapted to a 7.0 × 5.0 × 1.0 m^3^ aquarium with a water temperature of 24.5 ± 1.0 °C, pH 7.8, and a dissolved oxygen concentration of 9.2 ± 0.5 mg O_2_/L dechlorinated, aerated water. The fish were fed twice daily, at 07:00 and 17:00.

### Stress experiments

The stress experiments were performed as in our previous study [28]. A total of 180 fish were randomly divided into three tanks, each tank containing 60 fish. These tanks were shaken once every 5 min to simulate the transportation process. The death rates were calculated at 0, 2, 4, 6, and 8 h after transport. The mean length of the fish (*n* = 180) used in this experiment was 230.98 mm ± 9.26 (± standard error of the mean, SEM) and their mass was 70.28 g ± 5.76. These samples were used to analyze the association between *Glo1* gene alleles and stress, in a reverse transcription (RT)-quantitative PCR (qPCR) analysis of their *Glo1* mRNA expression profiles, and in western blotting, as described below.

Before sampling, the fish were euthanized with 70 mg/L buffered tricaine methanesulfonate (MS-222). The euthanized fish were immediately submerged in crushed ice to retard the degradation of their RNA. Tissue (brain) samples were stored at − 80 °C until later analysis. Total RNA was isolated by RNAiso Plus (Takara, Dalian, China) according to the manufacturer’s instructions, and the cDNA was synthesized, and qPCRs performed as described below.

To study the regulatory effects of salt ions on *Glo1* expression, seven groups of stress experiments were designed. Sea salt, NaCl, KCl, MgCl_2_, CaCl_2_, or sorbitol (1.0% each) was added to the culture water, and each test group contained three replicates, with 30 random fish per replicate. The no-salt group was designated the control group. The stress experiments were performed as described above. The brains of the fish were sampled at 0 and 4 h after transport (as described above), and RT-qPCR analysis of *Glo1* mRNA expression profiles were determined, and western blotting was performed as described below.

To study the regulatory effects of different concentrations of NaCl on *Glo1* expression, six groups of stress experiments were designed. No NaCl control, 0.5, 1.0, 1.5, and 2.0% NaCl were added to the culture water, and each test group contained three replicates, with 30 random fish per replicate. The no-salt group was designated the control group. The stress experiments were performed as described above. The brains of the fish were sampled at 0 and 4 h after transport (as described above), and RT-qPCR analysis of *Glo1* mRNA expression profiles were determined and western blotting was performed as described below.

### RT-qPCR analysis of *Glo1* mRNA expression profiles

For the *Glo1* mRNA expression analysis, total RNAs from five fish in each group were extracted from the brains of *C. nasus* with RNAiso Plus (Takara, China). The first-strand cDNA was synthesized with the ReverTra Ace® qPCR RT Kit (Toyobo, Osaka, Japan), and RT-qPCR was used to determine the *Glo1* expression profiles, using β-actin (*actb*) as the reference gene. The RT-qPCR primers 40S/40A for *Glo1* and B1/B2 for β-actin (Table 4) shared similar melting temperatures (Tm) and were designed to amplify 91-bp and 136-bp fragments, respectively. RT-qPCR was performed on the ABI 7500 Real-Time PCR System (ABI, Foster City, CA, USA) using 2× SYBR green real-time PCR mix (Takara, Japan). PCR amplification was performed in five samples in each group with each sample in triplicate, using the following cycling parameters: 94 °C for 2 min; followed by 40 cycles of 15 s at 94 °C, 15 s at 60 °C, and 45 s at 72 °C. All samples were analyzed in triplicate and the expressions of the target genes were calculated as the relative fold change, using the 2^−ΔΔCT^ method. One-way ANOVA followed by the Bonferroni post hoc test was used to analyze differences among all treatments. A probability (P) value < 0.05 was considered statistically significant.

**Table 4:** Sequences of primers used in this study

**Primer**	**Sequence**	**Usage**
**G1S**	CCACGCCTTACTGAAGCAGGCAAG	*Glo1* amplification
G1A	CCTGAGGTCTAAATCACCTG	
G2S	GCCAGGACTAGCCAAATTC	*Glo1* amplification
G2A	CAGCTGGCACTCACCT	
G3S	TACACACGAATCCTTGGAATG	*Glo1* amplification
G3A	GTGGAGTGGAGTTGCCTCCGCTG	
G4S	CTAGGAAGAGGCTACCTTTGGC	*Glo1* amplification
G4A	CTTGCAGGCAGCATACACATCTG	
G5S	TGCTGTATTGCTCCTGTTAC	Glo1 amplification
G5A	CTTGCAGGCAGCATACACATCTG	
G6S	GTTTGCTCTCTGTAGGCCACA	Glo1 amplification
G6A	CACATATCACCAGTTCTCGTTACC	
173S	CATTCCTCCAGAACCCCAGTAGTC	SNP genotyping
173A	TGTGTGGCACCAAAGCCTCTAGTT	
Glo1-F	AAGACAGCCTGGACCTTCTC	Probe amplification
Glo1-R	ACGTGGGTCTGAGTTTCCAT	
40S	ACATGGTGTCCATCTGCTCGTC	Glo1 RT-qPCR
40A	TCGTTACCCTCTCCCACTAGTTTTT	
B1	AACGGATCCGGTATGTGCAAAGC’	Beta-actin RT-qPCR
B2	GGGTCAGGATACCTCTCTTGCTCTG	

### Western blotting

The brain proteins of *C. nasus* were extracted with the KeyGEN Whole Cell Lysis Assay Kit (KeyGEN, Nanjing, China) and the protein content was determined with a bicinchoninic acid method kit (BCA Protein assay Kit; Pierce, Bonn, Germany). The other procedures were as described in our previous study [28].

### Association analysis of *Glo1* gene alleles and stress

The whole sequence of the *Glo1* gene was amplified with six pairs of gene-specific primers (Table 4). The amplicons ranged from 572 bp to 694 bp in length. Each PCR was performed in an ABI Thermal Cycler (ABI) in a 25 μL reaction volume containing 100 ng of the DNA template. The annealing temperatures were calculated with the Primer Premier 5.0 software (http://www.premierbiosoft.com/primerdesign/index.html). The PCR products from 30 dead fish and 30 surviving fish were purified and sequenced with the ABI 3730 DNA Analyzer (ABI). The polymorphic loci were detected from a sequence alignment of different individuals using Vector NTI Suite 11.0 (Invitrogen, Carlsbad, CA, USA).

To genotype these SNPs with PCR and Sanger sequencing, an additional pair of primers (173S/173A) was designed (Table 4). PCR was performed with all the samples of dead and surviving fish. The resulting fragments were separated on 1.0% agarose gels and purified with the Axygen DNA Gel Extraction Kit (Axygen, Union City, CA, USA). The purified fragments were then sequenced (Tianlin, Wuxi, China).

We estimated the allele and genotype frequencies and analyzed their associations with stress tolerance using R3.3.3 (https://cran.r-project.org/bin/windows/base/old/3.3.3/). To further test the associations between the SNPs and stress tolerance, we tested the linkage disequilibrium based on the genotyping results. The loci in strong linkage disequilibrium were selected for a haplotype analysis with the SHEsis software (http://analysis.bio-x.cn/SHEsisMain.htm). The χ^2^ test was used to test the significance of differences. *P* values less than 0.05 were considered statistically significant.

### Southern blotting

Southern blotting was used to determine the number of copies of the *Glo1* gene in the *C. nasus* genome, as described previously [41]. Briefly, genomic DNA from the fish brain was isolated with TIANamp Marine Animals DNA kit (Tiangen, Beijing, China), and the DNA concentrations in the samples were adjusted to 100 ng/μL. The genomic DNAs were digested with enzymes *Bam*HI, *Kpn*I, and *Hin*dIII. The digested DNA was separated with 0.8% agarose gel electrophoresis. DNA fragments were transferred to a positively charged nylon membrane (Millipore, Boston, MT, USA) and then hybridized with a digoxigenin-labeled *Glo1*-specific probe (the probe was amplified with PCR and labeled with digoxigenin using the PCR primers Glo1-F/Glo1-R, shown in Table 4). An NBT/BCIP color detection kit (DIG High Prime Lab/Detection K1 kit; Roche, Basel, Germany) was then used to detect the fragments.

### Analysis of nucleotide and amino acid sequences

The nucleotide and predicted amino acid sequences of *Glo1* were analyzed using the DNA Figures software (http://www.bio-soft.net/sms/index.html). The similarities between *Glo1* from *C. nasus* and the *Glo1* genes of other organisms were analyzed using the BLASTP search program (http://www.ncbi.nlm.nih.gov/blast). The *C. nasus* Glo1 amino acid sequence was compared with those of other species with ClustalX 1.83 (http://www.ebi.ac.uk/clustalW/) and GeneDoc (http://www.nrbsc.org/gfx/genedoc/). A phylogenetic tree was constructed using MEGA 3.1 (http://megasoftware.net; Table 5). The three-dimensional structure of the Glo1 protein was predicted with the SWISS-MODEL online software (https://swissmodel.expasy.org/).

**Table 5:** GenBank accession numbers of the *Glo1* sequences used in this study

**Species**	**Accession no.**
**DNA sequences**	
*Canis lupus familiaris*	NC_006594.3
*Mus musculus*	NC_000083.6
*Gallus gallus*	NC_006090.3
*Xenopus tropicalis*	NW_004669463.1
*Larimichthys crocea*	NW_017608179.1
*Pygocentrus nattereri*	NW_016243793.1
*Cyprinus carpio*	LHQP01009933.1
*Danio rerio*	NC_007124.5
*Sinocyclocheilus anshuiensis*	NW_015557379.1
*Oryza sativa*	NC_008398.2
*Anopheles gambiae*	NT_078267.5
Kluyveromyces lactis	NC_006042.1
*Saccharomyces cerevisiae*	NC_001145.3
Schizosaccharomyces pombe	NC_003423.3
*Magnaporthe oryzae*	NC_017852.1
*Neurospora crassa*	NW_001849812.1
**Protein sequences**	
*Homo sapiens*	NP_006699.2
*Pan troglodytes*	XP_001173775.1
*Macaca mulatta*	XP_001117098.1
*Canis familiaris*	XP_532129.3
*Bos taurus*	NP_001076965.1
*Mus musculus*	NP_001107032.1
*Rattus norvegicus*	NP_997477.1
*Gallus gallus*	XP_419481.1
*Danio rerio*	NP_998316.1
*Astyanax mexicanus*	XP_007238567.1
*Osmerus mordax*	ACO09023.1
*Anoplopoma fimbria*	ACQ58210.1
*Salmo salar*	ACH70673.1
*Neolamprologus brichardi*	XP_006779779.1
*Maylandia zebra*	XP_004539831.1
*Haplochromis burtoni*	XP_005913134.1
*Poecilia formosa*	XP_007549146.1
*Oryzias latipes*	XP_004067520.1
*Oreochromis niloticus*	XP_003437619.1
*Poecilia reticulata*	XP_008403069.1

### Analysis of immune inflammation and antioxidant capacity

TNF-α, cytochrome c, caspase-9, and caspase-3 were analyzed using an enzyme-linked immunosorbent assay kit (Zhaorui, Shanghai, China), as described by the manufacturer. Total antioxidant capacity (T-AOC), superoxide dismutase (SOD), catalase (CAT), and glutathione peroxidase (GSH-Px) were examined using appropriate detection kits according to the manufacturer’s instructions (Nanjing Jiancheng Chemical Industrial, Nanjing, China).

One-way ANOVA followed by the Bonferroni post hoc test was used to analyze differences among all treatments. A probability (P) value < 0.05 was considered statistically significant.

### Glo1 expression and survival analysis in cancers

The expression data of GLO1 in pan-cancer were extracted from TCGA database (http://cancergenome.nih.gov) and the GTEX database (https://www.gtexportal.org/home/). Statistical analysis of the differences in expressions were performed using GraphPad Prism 6, with no special comments. Student’s *t*-test was used to compare the difference between two groups. Overall survival was shown as a Kaplan-Meier curve, which was calculated using the log-rank test. A value of *p* < 0.05 was considered statistically significant. R/Bioconductor survival and the Survminer package were used for survival analyses of GLO1 in pan-cancer.

## Abbreviations

CAT: catalase
Glo1: glyoxalase 1
GSH-Px: glutathione peroxidase
RT-qPCR: reverse transcription-quantitative PCR
SNP: single-nucleotide polymorphisms
SOD: superoxide dismutase
T-AOC: total antioxidant
Tm: melting temperature

## References

[1] Chen T. T., Jiang T., Liu H. B., Li M. M., Yang J. (2017). Do all long supermaxilla-type estuarine tapertail anchovies (Coilia nasus Temminck et Schlegel, 1846) migrate anadromously?. Journal of Applied Ichthyology.

[2] Yang J, Jiang T, Liu H (2011). Are there habitat salinity markers of the Sr:Ca ratio in the otolith of wild diadromous fishes? A literature survey. Ichthyol Res.

[3] Du F, Xu G, Nie Z, Xu P, Gu R (2014). Transcriptome analysis gene expression in the liver of Coilia nasus during the stress response. BMC Genomics.

[4] Schwartz PJ, Volders PGA (2014). Sudden death by stress. J Am Coll Cardiol.

[5] Gale CP: Characterisation and functional analysis of the human glyoxalase-1 gene. University of Leeds*;*2003. 10.1016/j.gene.2004.07.009 15475166

[6] Rabbani N, Thornalley PJ (2011). Glyoxalase in diabetes, obesity and related disorders. Semin Cell Dev Biol.

[7] Yadav SK, Singlapareek SL, Sopory SK (2008). An overview on the role of methylglyoxal and glyoxalases in plants. Drug Metab Drug Interac.

[8] Thornalley PJ (2003). Glyoxalase I--structure, function and a critical role in the enzymatic defence against glycation. Biochem Soc Trans.

[9] Chakraborty S, Gogoi M, Chakravortty D (2015). Lactoylglutathione lyase, a critical enzyme in Methylglyoxal detoxification, contributes to survival of Salmonella in the nutrient rich environment. Virulence.

[10] Chocholatý M, Jáchymová M, Schmidt M, Havlová K, Křepelová A, Zima T, Babjuk M, Kalousová M (2015). Polymorphisms of the receptor for advanced glycation end-products and glyoxalase I in patients with renal cancer. Tumor Biol.

[11] Chen F, Wollmer MA, Hoerndli F, Münch G, Kuhla B, Rogaev EI, Tsolaki M, Papassotiropoulos A, Götz J (2004). Role for glyoxalase I in Alzheimer's disease. Proc Natl Acad Sci U S A.

[12] Hambsch B (2011). Altered glyoxalase 1 expression in psychiatric disorders: cause or consequence?. Semin Cell Dev Biol.

[13] Hovatta I, Tennant RS, Helton R, Marr RA, Singer O, Redwine JM, Ellison JA, Schadt EE, Verma IM, Lockhart DJ (2005). Glyoxalase 1 and glutathione reductase 1 regulate anxiety in mice. Nature.

[14] Kuhla B, Boeck K, Lüth HJ, Schmidt A, Weigle B, Schmitz M, Ogunlade V, Münch G, Arendt T (2006). Age-dependent changes of glyoxalase I expression in human brain. Neurobiol Aging.

[15] Kuhla B, Boeck K, Schmidt A, Ogunlade V, Arendt T, Münch G, Lüth HJ (2007). Age- and stage-dependent glyoxalase I expression and its activity in normal and Alzheimer's disease brains. Neurobiol Aging.

[16] Rabbani N, Thornalley PJ (2012). Methylglyoxal, glyoxalase 1 and the dicarbonyl proteome. Amino Acids.

[17] Distler MG, Plant LD, Sokoloff G, Hawk AJ, Aneas I, Wuenschell GE, Termini J, Meredith SC, Nobrega MA, Palmer AA (2012). Glyoxalase 1 increases anxiety by reducing GABAA receptor agonist methylglyoxal. J Clin Investig.

[18] Lin CC, Yin MC (2010). Antiglycative and anti-VEGF effects of s-ethyl cysteine and s-propyl cysteine in kidney of diabetic mice. Mol Nutr Food Res.

[19] Santel T, Pflug G, Hemdan NYA, Schäfer A, Hollenbach M, Buchold M, Hintersdorf A, Lindner I, Otto A, Bigl M (2008). Correction: Curcumin inhibits Glyoxalase 1—a possible link to its anti-inflammatory and anti-tumor activity. PLoS One.

[20] Kim KM, Kim YS, Jung DH, Lee J, Kim JS (2012). Increased glyoxalase I levels inhibit accumulation of oxidative stress and an advanced glycation end product in mouse mesangial cells cultured in high glucose. Exp Cell Res.

[21] Du F, Xu G, Gao J, Nie Z, Xu P, Gu R (2016). Transport-induced changes in hypothalamic–pituitary–interrenal axis gene expression and oxidative stress responses in Coilia nasus. Aquac Res.

[22] Lander ES, Linton LM, Birren B, Nusbaum C, Zody MC, Baldwin J, Devon K, Dewar K, Doyle M, FitzHugh W (2001). Initial sequencing and analysis of the human genome. Nature.

[23] Morgenstern J, Fleming T, Schumacher D, Eckstein V, Freichel M, Herzig S, Nawroth P (2017). Loss of Glyoxalase 1 induces compensatory mechanism to achieve dicarbonyl detoxification in mammalian Schwann cells. J Biol Chem.

[24] Zimin AV, Cornish AS, Maudhoo MD, Gibbs RM, Zhang X, Pandey S, Meehan DT, Wipfler K, Bosinger SE, Johnson ZP (2014). A new rhesus macaque assembly and annotation for next-generation sequencing analyses. Biol Direct.

[25] Skow LC, Womack JE, Petresh JM, Miller WL (1988). Synteny mapping of the genes for 21 steroid hydroxylase, alpha a crystallin, and class I bovine leukocyte antigen in cattle. DNA.

[26] Stratmann Bernd, Goldstein Bernhard, Thornalley Paul, Rabbani Naila, Tschoepe Diethelm (2017). Intracellular Accumulation of Methylglyoxal by Glyoxalase 1 Knock Down Alters Collagen Homoeostasis in L6 Myoblasts. International Journal of Molecular Sciences.

[27] Wang S, Yang Q, Wang Z, Feng S, Li H, Ji D, Zhang S (2017). Evolutionary and expression analyses show co-option of khdrbs genes for origin of vertebrate brain. Front Genet.

[28] Du F, Xu G, Li Y, Nie Z, Xu P (2016). Glyoxalase 1 gene of Coilia nasus : molecular characterization and differential expression during transport stress. Fish Sci.

[29] Lushchak VI (2011). Environmentally induced oxidative stress in aquatic animals. Aquat Toxicol.

[30] Atkinson S, Crocker D, Houser D, Mashburn K (2015). Stress physiology in marine mammals: how well do they fit the terrestrial model?. J Comp Physiol B, Biochem Syst Environ Physiol.

[31] Lópezolmeda JF, Blancovives B, Pujante IM, Wunderink YS, Mancera JM, Sánchezvázquez FJ (2013). Daily rhythms in the hypothalamus-pituitary-Interrenal Axis and acute stress responses in a teleost flatfish, Solea senegalensis. Chronobiol Int.

[32] Stratholt ML, Donaldson EM, Liley NR (1997). Stress induced elevation of plasma cortisol in adult female coho salmon ( Oncorhynchus kisutch ), is reflected in egg cortisol content, but does not appear to affect early development. Aquaculture.

[33] Comline RS, Edwards AV, Nathanielsz PW (1970). The effects of cortisol on the carbohydrate metabolism of hypophysectomized and of thyroidectomized calves. J Physiol.

[34] Leach GJ, Taylor MH (1982). The effects of cortisol treatment on carbohydrate and protein metabolism in Fundulus heteroclitus. Gen Comp Endocrinol.

[35] Brouwers Olaf, Niessen Petra M. G., Miyata Toshio, Østergaard Jakob A., Flyvbjerg Allan, Peutz-Kootstra Carine J., Sieber Jonas, Mundel Peter H., Brownlee Michael, Janssen Ben J. A., De Mey Jo G. R., Stehouwer Coen D. A., Schalkwijk Casper G. (2013). Glyoxalase-1 overexpression reduces endothelial dysfunction and attenuates early renal impairment in a rat model of diabetes. Diabetologia.

[36] Santarius T, Bignell GR, Greenman CD, Widaa S, Chen L, Mahoney CL, Butler A, Edkins S, Waris S, Thornalley PJ (2010). GLO1-a novel amplified gene in human cancer. Genes Chromosomes Cancer.

[37] Fujimoto M, Uchida S, Watanuki T, Wakabayashi Y, Otsuki K, Matsubara T, Suetsugi M, Funato H, Watanabe Y (2008). Reduced expression of glyoxalase-1 mRNA in mood disorder patients. Neurosci Lett.

[38] Distler MG, Palmer AA (2012). Role of Glyoxalase 1 (Glo1) and methylglyoxal (MG) in behavior: recent advances and mechanistic insights. Front Genet.

[39] Collignon E, Canale A, Al Wardi C, Bizet M, Calonne E, Dedeurwaerder S, Garaud S, Naveaux C, Barham W, Wilson A, Bouchat S, Hubert P, Van Lint C, Yull F, Sotiriou C, Willard-Gallo K, Noel A, Fuks F (2018). Immunity drives regulation in cancer through NF-κB. Sci Adv.

[40] Courtnay R, Ngo DC, Malik N, Ververis K, Tortorella SM, Karagiannis TC (2015). Cancer metabolism and the Warburg effect: the role of HIF-1 and PI3K. Mol Biol Rep.

[41] Southern E (2006). Southern blotting. Nat Protoc.

